# Temporal Check-All-That-Apply (TCATA) Reveals Matrix Interaction Effects on Flavor Perception in a Model Wine Matrix

**DOI:** 10.3390/foods8120641

**Published:** 2019-12-04

**Authors:** Andrew R. Poveromo, Helene Hopfer

**Affiliations:** 1 Department of Food Science, The Pennsylvania State University, State College, PA 16802, USA; arp67@psu.edu; 2 Sensory Evaluation Center (SEC), The Pennsylvania State University, State College, PA 16802, USA

**Keywords:** temporal sensory evaluation, model wine, sensory perception, matrix interactions

## Abstract

Traditionally, the sensory properties of wine were characterized using a trained panel and descriptive analysis (DA)—a static sensory evaluation method. As wine is a complex mixture, with evolving sensory properties, a way to capture these changes is needed in order to fully describe the sensory experience of wine perception. In this study, temporal check-all-that-apply (TCATA), a dynamic sensory evaluation method, was used to characterize model wine samples reminiscent of a white, hybrid wine. Twelve model wines varied in levels of ethanol, glycerol, and caffeic acid, representing commercial levels in Pennsylvania. Samples were evaluated for up to three minutes by a trained TCATA panel (*n* = 12) for flavor, taste, and mouthfeel attributes. In general, the experimental factors, ethanol and glycerol, along with interactions between factors, had the greatest temporal effects, with significant differences in flavor attributes occurring within the first 30 s of evaluation, while taste and mouthfeel attributes showed significant differences throughout the evaluation period. Overall, ethanol had the greatest impact on temporal wine perception. The findings of this study further suggest that a temporal evaluation method, like TCATA, should be paired with DA to completely characterize a complex and evolving sample. Further, changes in wine matrix components affect sensory perception both in direct and indirect ways—the latter indicated by taste-taste suppression and cross-modal interaction effects.

## 1. Introduction

Complex mixtures, such as wine, have shown considerable mixture effects. Both non-volatile and volatile components of the wine matrix are affecting each other through interactions. Further, these interactions affect wine perception on multiple levels: (i) on a chemical level, it has been shown that matrix components affect the partitioning of certain aroma compounds into the headspace [1]; (ii) on a perceptual level, it is known that taste sensations often suppress each other, while cross-modal interactions can lead to antagonistic or synergistic behavior [2].

In addition, major matrix components like ethanol as well as non-volatile phenolic compounds and glycerol all have been shown to affect wine flavor: ethanol has been shown to decrease citrus aroma and increase bitter taste in white wines [3], and phenolic compounds (i.e., hydroxycinnamates, flavanols, and flavan-3-ols) have been shown to increase astringency, viscosity, hot mouthfeel, and bitterness [4], while glycerol has been shown to increase volatile compound partitioning in situations where ethanol is absent; this change in partitioning has been shown to affect aroma perception [1].

Further, these factors do not act in isolation, but rather interactively with each other as well as with aroma compounds [5]. For example, both C13-norisoprenoids (e.g., *β*-damascenone and *β*-ionone) and dimethyl sulfide have been independently shown to enhance fruity aromas, while ethanol suppresses them [6].

The effect of matrix components on wine sensory perception has mostly been studied using DA, where a trained sensory panel makes qualitative and quantitative single point measurements of sensory attributes. While this approach provides valuable information, wine matrix factors have also been shown to affect wine sensory attributes in a dynamic fashion—that is, the perception of a sensory attribute changes from one moment to another, e.g., during the duration of a single or multiple sips [7]. These dynamic changes are caused by multiple product- as well as human-inherent factors, for example, chewing and breathing, and differences in individual saliva composition [8,9]. As wine is exposed to oxygen, chemical reactions taking place have been shown to change the sensory perception [10]. Therefore, studying these temporal sensory changes is critical to understand what compositional factors are driving the overall sensory experience during wine consumption.

In the recent past, two major temporal methods have been applied to dynamically assess wines, namely, temporal dominance of sensation (TDS) and temporal check-all-that-apply (TCATA) [7,11,12,13,14,15,16]. In TDS, participants are given a list of product-relevant sensory attributes and are asked to select the most “dominant” attribute at any time, the one that “catches the attention at a given time”, not necessarily the one that is the most intense [15]. Comparing DA to TDS, it was found that TDS should be paired with DA for precise measurements as DA does not capture temporal aspects and TDS does not provide intensity ratings [11], and that while TDS cannot replace a complete sensory profile, it is easy to use and particularly useful for products with a “weak sensory difference” [7]. Work by Frost et al. confirmed that TDS captures the sensory aspects of wines that are not captured by DA or instrumental methods [14,15].

While TDS, as a temporal evaluation method, has been shown to elucidate differences in dynamic vs. static wine flavor perception, the major limitation of this method is that it only provides information on the most dominant attribute. For a complex product like wine, focusing on the dominant attribute over time may not be enough, as more than one attribute may be present and, thus, needs to be captured to understand its contribution to overall flavor [17]. TCATA overcomes this limitation, as it is an extension of the commonly used check-all-that-apply (CATA) method: participants are provided with an attribute list salient for a particular product and asked to select any of the listed attributes that they perceive over the period of evaluation (e.g., a single sip). While the collected frequency data does not necessarily equate to intensity ratings, Campo et al. showed for Pinot Noir wines that attributes selected most often in CATA were also rated highest in intensity using classical DA [18]. Comparing TDS to TCATA, Ares et al. [17] and Meyners and Castura [19] concluded that TCATA may lead to better product differentiation, as more than one attribute at a time can be checked. However, both methods were found to be complementary to each other.

Past studies looked at the temporal effects of major matrix factors in wine: ethanol, for example, was shown to affect both bitter taste intensity and duration over the period of a single sip, leading to both increased bitter intensity and duration with increasing ethanol content in German white wines [11]. Using TDS, the authors showed that bitter taste persistence, an aspect that was not picked up by the DA panel, was able to explain the differences between the wines. Additionally, they were able to link bitter taste persistence over the duration of a sip to not only ethanol, but also to changes in fructose concentration. TDS revealed that fructose inversely affected the duration of bitter dominance more than ethanol, in that increasing levels of fructose led to a shorter duration of bitter dominance [11].

Similar results were seen in red Syrah wines, where higher levels of ethanol not only increased perceptions of warmth, astringency, bitter taste, spice and dark fruit flavors, but also led to a longer total duration of wine finish (88 vs. 76 s for the low-alcohol wines), as assessed by TCATA [12]. In contrast, the low-alcohol wines were characterized by high red fruit perception during the first part of evaluation, followed by high citation levels of green flavors in the middle part, and a lasting sour taste towards the end of the evaluation of a single sip [12]. The reported flavor changes over time indicated that ethanol is affecting flavor attributes that are not directly tied to the perception of ethanol in indirect ways, through cross-modal interactions and/or taste-taste suppression.

The effects of carbonation levels in sparkling wines were similarly studied with TCATA. Carbonation is an important quality attribute of sparkling wine, and the authors found that lowering carbonation (i.e., less CO_2_ present in the wine) resulted in muted perception for all sensory attributes throughout the entire evaluation period, except for bitter and sour taste, which were unaffected by carbonation. For the highest level of carbonation, the duration of numbing and tingling sensations increased from 46 to 65 s, and interestingly, for the high carbonation treatment (7.5 g/L CO_2_), an increase in sour taste between 45 and 60 s was observed [13]. This is interesting as sourness was initially (0–45 s) higher in the lower carbonation treatments but flipped after that—an observation that was only made due to the use of a temporal sensory evaluation method.

It was found that combining static DA with a temporal assessment allows for a comprehensive understanding of wine flavor perception [14,15]. For example, the effects of changing tannins, acidity, and ethanol levels in red Merlot wines not only led to significant flavor differences as evaluated by DA, but also changed aroma, flavor, taste, and mouthfeel attributes to a different degree over time (90 s) [15]. For example, varying tannin concentration either to a high or low level (over 1000 vs. ~725 mg/L catechin equivalents), in combination with pH (5, 6, and 7) and ethanol (14%, 14.5%, and 15% *v/v*), affected sour taste, bitter taste, and astringent mouthfeel. While static DA revealed that sour taste increased with increasing levels of acidity, TDS additionally found that tannin concentration also affected sour taste perception at the beginning of evaluation (0–15 s), where sourness was higher in the higher tannin samples. Using TDS, bitter taste was found to not be the dominant attribute at the end of evaluation (~the final 15 s) for the high tannin samples, while it was dominant at the end of evaluation for the low tannin samples. This indicates the suppressing effects of bitter and sour taste [20]. Last, for astringency, increased tannin concentration led to increased astringency in the DA, and TDS indicated that this increase could be explained by astringency being perceived earlier. This implies that for astringency, the onset and duration of the sensation seems to drive the increased DA ratings. The authors also confirmed that TDS captures sensory aspects of wines that are not captured by DA or instrumental methods [14].

Despite the information on how different wine matrix components (e.g., ethanol, CO_2_, etc.) affect temporal flavor perception of wines, it is unclear how these matrix components interact with each other and aroma compounds and affect flavor perception over time in a white wine matrix. Thus, in this study, model white wine samples were evaluated by TCATA to assess the dynamic changes in sensory perception as a function of varying wine matrix components, such as ethanol, glycerol, and phenolics. The objective of this study was to identify the factors that significantly affect the temporal perception of a white, hybrid model wine. Based on prior literature, we hypothesized that TCATA would identify different experimental factors than DA that are responsible for observed temporal sensory differences. Further, we hypothesized that ethanol content would lead to the greatest changes in temporal sensory perception, both in duration and citation frequency.

## 2. Materials and Methods

### 2.1. Model Wine Samples

For this study, 12 different model wine samples were used (Table 1), varying in levels of ethanol, caffeic acid, and glycerol, while keeping pH, titratable acidity, protein concentration, and aroma concentration constant. The levels of ethanol (200 proof, Decon Labs, King of Prussia, PA) and glycerol (>99%, Sigma-Aldrich, St. Louis, MO, USA) were chosen to cover the ranges observed in Pennsylvanian white wines. The greatest phenolic fraction in white wines are hydroxycinnamates [21], found in white wines at around 64 mg/L (range 17–130 mg/L) [22]. The hydrocinnamate caffeic acid (>98%, Sigma-Aldrich, St. Louis, MO, USA) was selected as the model phenolic compound, as it is safe for human consumption [22,23] and found in wines in the range of 2.4–40 mg/L [24]. Additionally, the samples also contained 0.5 g/L bovine serum albumin (BSA) (>98%, Sigma-Aldrich, St. Louis, MO, USA). Although BSA, with a size of around 66 kDa, is about double the size of typical wine proteins [21], it has previously been used as a model protein for wine research [8], as it is safe for human consumption [25,26,27]. Potassium hydrogen tartrate (99%, Spectrum Chemical, Gardena, CA, USA) was added to achieve a titratable acidity of 7.5 g/L and a pH of 3.5. Last, the same level of an aroma fraction (Table 1b) was added to all model wine samples. The composition was based on a 2017 research Traminette wine [28] and Chardonnay [3].

For sample production, reverse osmosis water was combined with ethanol and potassium hydrogen tartrate in 5 gallon buckets, which were aliquoted and mixed with the remaining components, except for the aroma fraction. Model wines were measured into 500 mL glass canning jars (Ball, Broomfield, CO, USA) and stored at 4 °C until the day of sensory training or testing (max. 6 weeks). One milliliter of the aroma fraction was added to the 500 mL samples 5 h prior to use.

### 2.2. Sensory Evaluation by Temporal Check-All-That-Apply (TCATA)

A trained panel of 12 participants (8 females and 4 males, aged 23–65) was recruited for this study from the participant database of the Sensory Evaluation Center (SEC) at Penn State, based on prior participation in trained panels or involvement in grape and wine research. Panelists were screened for availability, white wine consumption (min of 2–3 times/month), food allergies, health concerns, and taste or smell defects. Penn State’s IRB reviewed and oversaw the study (STUDY00010095).

On the day of training and testing, 1 mL of the aroma fraction was added to 500 mL model wine samples. Twenty milliliters were poured approximately 1 h before evaluation into clear ISO wine glasses and covered with a disposable, polystyrene Petri dish (60 × 15 mm, VWR International, Radnor, PA). Wine glasses were labeled with a randomized three-digit blinding code on the stem.

Panelists received three, 90 min training sessions, where they were familiarized with the basic taste, mouthfeel, and flavor references (Table 2). Attributes were selected based on a prior DA on the same set of wines [29], and included three flavor (apple, pear, citrus), three basic taste (sweet, sour, bitter), and two mouthfeel (astringent, warm/hot) attributes. An “other” option was also provided to prevent potential “dumping” bias [30].

Panelists also received training on the TCATA method through multiple methods. First, they completed a guided practice example of TCATA, as provided by Compusense (Compusense Cloud, Academic Consortium, Guelph, ONT, CA). Panelists were instructed to imagine that they were tasting a cherry flavored beverage. After starting the timer, instructions popped up on the screen telling them what sensory attributes they were perceiving and when to select or deselect them. Panelists were allowed to complete this exercise as many times as they wished.

In the next training session, a selection of music was played (“Somebody to Love” by Queen). This song was selected because it incorporated multiple instruments and vocals—many of which are present simultaneously. When the music began, panelists started their timer and selected the different elements that they heard in the music (e.g., “lead vocals”, “backup vocals”, “guitar”, “piano”, “drums”). After familiarizing themselves with the method, panelists began practicing evaluating the model wine samples using TCATA.

Training success was assessed by (i) having panelists evaluate blind duplicate samples in a consistent and similar manner, and (ii) correctly identifying the references that they had been trained on. To assess training success, the difference curves of two different duplicate samples were checked; as no significant differences between the blind duplicates were found, the panel was deemed trained.

Following training, the 12 white, hybrid model wine samples were evaluated in three sensory replicates in six 60 min evaluation sessions. Evaluation was conducted in individual booths at room temperature under white light. Each panelist evaluated six model wine samples in each individual evaluation session. A modified Williams-Latin square design was created so that panelists saw the same six wines in any given evaluation session but counterbalanced in order within a session.

For the sample evaluation, panelists were first instructed to take a sip of water to cleanse their palate. Panelists then took a sip of the sample and started the onscreen timer. They were instructed to begin selecting and deselecting attributes immediately after starting the timer (no auto-deselect option). After 10 s of moving the wine sample around in their mouth, they were instructed to expectorate. This 10 s time period was discussed during the training and panelists decided that they needed no more time than that; similar in-mouth evaluation periods (10–15 s) have been used previously for wine assessment with TCATA and TDS [12,13,14]. The evaluation of the sample continued for up to three minutes. Panelists were not allowed to stop the timer early, but they could deselect all attributes if they were no longer perceiving any of them.

In between individual samples, panelists had a forced 60 s break, during which they were encouraged to cleanse their palate with deionized water and unsalted soda crackers (Mondeléz Global LLC, Deerfield, IL, USA). Additionally, after the third sample (halfway through an evaluation session), panelists had a forced 10 min break to avoid fatiguing. During this break, panelists were encouraged to leave the test booth and walk around.

All sensory data were collected using Compusense Cloud software.

### 2.3. Statistical Analysis

All statistical analysis was conducted using RStudio (ver. 1.1.383, Boston, MA, USA) and R software (ver. 3.4.3, R Foundation for Statistical Computing, Vienna, Austria), with the additional packages *tempR* (ver. 0.9.9.12) [31] and *ggplot2* (ver. 2.2.1) [32]. Statistical significance was defined as *p* ≤ 0.05 for all analyses.

Initial analyses assessed panelist and panel agreement, as suggested by [19,33,34], to ensure that panelists agreed with each other, and with themselves for the replicate samples. Panelist agreement with themselves was checked for all samples and attributes. Panelist agreement with the rest of the panel was checked for selected attributes (citrus flavor, sweet taste, and warm/hot mouthfeel) and samples, to cover all different sensory modalities and include high, medium, and low intensity attributes. Selected samples were 14% and 10% ethanol levels with low levels of caffeic acid (20 mg/L) and glycerol (5 g/L).

For panelist agreement, the similarity between one panelist and the rest of the panel was quantified by comparing a matrix of TCATA data for one panelist to a matrix of TCATA data for the rest of the panel, and expressed as an index between 0 and 1, with 1 indicating perfect similarity. Second, panelist consistency across the three sensory replicates was quantified as the similarity between repeated evaluations of a single panelist, and expressed as an index between 0 and 1, with 1 indicating perfect panelist consistency.

Data resulting from a TCATA study is binary, meaning that it is a series of zeros and ones for each attribute, sensory replicate, and panelist. Every second where an attribute is selected gets marked as a one. If the attribute is not selected, it gets marked as a zero. To assess the effects of our experimental factors—ethanol, glycerol, and caffeic acid levels, the TCATA data was treated as count data. This approach is similar to a prior study where proportions of selections were used for data analysis by calculating the proportion of panelists who selected an attribute at any given moment during evaluation [13]. The authors used citation proportions for each sensory attribute for every 0.1 s, which was their selected time segmentation. Similarly, to test which experimental factors (ethanol, glycerol, and caffeic acid) and interactions significantly affect the sensory attributes throughout the three-minute evaluation period, Analysis of Variance (ANOVA) was run for each of the 9 attributes, using 30 s time bins. These 30 s time segments should be small enough to detect temporal changes during the 3 min evaluation period, but allow to test for significant differences due to the experimental factors without being affected by slightly different selection times of the individual panelists, similar to the procedures described by [35,36].

A trajectory principal component analysis (PCA) was created to visualize the temporal changes of each sample during the 3 min evaluation period. This type of PCA can be understood as the super-positioning of individual PCAs run at every time segment; the resulting lines, or trajectories, indicate how each sample changes throughout the evaluation period. Such a visual depiction of global changes allows one to follow and better understand the sensory changes of each sample throughout time as well as enables one to compare samples to each other. It is a very powerful and elegant visualization method that presents several thousands of data points into one easy-to-understand figure [13].

## 3. Results

### 3.1. Panelist Consistency and Panel Agreement

Assessments of panel consistency and panel agreement were first conducted to ensure data quality and validity. For panel agreement, three sensory attributes (citrus flavor, sweet taste, warm mouthfeel) and two different ethanol levels (10% and 14%) were selected, allowing for the testing of panel agreement for all three sensory modalities (retronasal aroma, basic taste, mouthfeel) and the most extreme samples, i.e., the ones one would expect to significantly differ. Second, the repeatability of each panelist was assessed to test whether panelists were consistent in their evaluations across the three sensory replicates.

Overall, the panelists were in good agreement with the rest of the panel, with agreement indices of 0.60 and higher (Table 3). In a previous study [34], agreement values ranged from 0.69 to 0.74, while median agreement values for this study were 0.71 and higher, except for Panelist 1, who showed the least agreement with the rest of the panel (values between 0.16 and 0.60). As participants are human subjects, there is always variability between people, and it is not unexpected that someone may stray from the group. However, as the majority of panelists were in high agreement, all data were kept for analysis, and no panelist was removed from the data.

Second, the repeatability index can take values between 0 and 1, where 0 is not repeatable and 1 is completely repeatable. As seen in Table 3, all panelists showed very high consistency across the three sensory replicates as repeatability indices ranged from 0.75 to 0.88. Overall, the TCATA panel showed both very high panel agreement and repeatability across sensory replicates, indicating high quality TCATA data.

### 3.2. Visualization of Temporal Sensory Perception of Model Wines Varying in Ethanol, Glycerol, and Caffeic Acid

**Figure 1:**
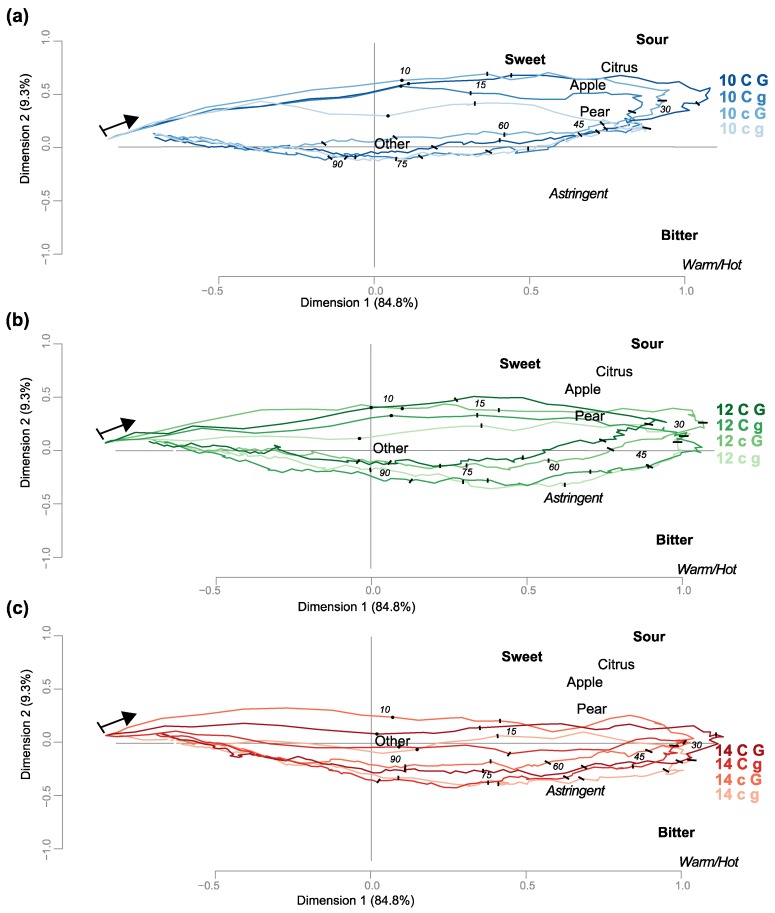
Principal component analysis (PCA) trajectories, separated by ethanol content. (**a**) 10% (*v/v*), (**b**) 12% (*v/v*), and (**c**) 14% (*v/v*) ethanol. Capital and lowercase letters represent high and low levels of the glycerol (G g) and caffeic acid (C c). Attributes, represented by black triangles, include flavors in regular, tastes in bold, and mouthfeels in italicized font. Expectoration at 10 s and time markers every 15 s are indicated in each trajectory curve.

To provide a visual overview of the observed temporal changes across the 12 model wines, varying in ethanol, caffeic acid, and glycerol concentrations, and to identify the major drivers of sensory perception over time, a trajectory principal component analysis (PCA) biplot was generated (Figure 1).

Plotting the first two dimensions of the resulting PCA, close to 95% of the total variance is captured, with the first principal component (PC 1) explaining 85%, thus, capturing most of the variability. It becomes apparent that PC 1 is capturing the temporal nature of the data: starting from the left-hand side of the plot, indicating the start of the evaluation, sample trajectories trend towards the right-hand side of the PCA plot before curling back towards the left as panelists reach the end of the evaluation period of 180 s and perceive less and less sensory attributes.

In Figure 1, samples are shown separated by ethanol levels, with time markers for the 10 s expectoration prompt and for every 15 s throughout evaluation. For most of the samples, the panelists perceived sweet and sour tastes, apple, citrus, and pear flavors fairly quickly (within the first 15–30 s) while bitter taste, and astringent and warm/hot mouthfeels were perceived later (45–60 s), as the latter attributes are located more towards the right and bottom side of the plot, close to the “backward” trajectories. Also apparent is a grouping of samples by ethanol content along the second principal component (PC 2), with the 14% ethanol samples positioned at the bottom (Figure 1c), the 10% ethanol samples on the top (Figure 1a), and the 12% ethanol samples in-between (Figure 1b). The PCA also shows that bitter taste and warm/hot mouthfeel are located on opposite ends along the second dimensions from sour taste, with highest citation frequencies for bitter and warm/hot in the samples with the highest ethanol levels.

Similarly, sweet taste was mostly associated with the lowest ethanol level samples and was positioned opposite from bitter taste on PC 2, indicating potential taste-taste suppression.

Looking at samples within an ethanol level (Figure 1a–c), one can see that the higher glycerol samples (G) are closer positioned to the fruity flavors of apple and pear as well as the sweet taste attribute than the lower glycerol samples (g). Further, this effect is apparent at all ethanol levels, where, even at the 14% ethanol concentration, the two samples with high levels of glycerol (14 CG and 14 cG) are both positioned closer to sweet taste and apple and pear flavor than both 14 Cg and 14 cg wines.

### 3.3. Analysis of Variance Reveals Compositional Drivers of Temporal Perception of Wine

To obtain a more detailed picture of the major drivers of the observed sensory changes over the evaluation period, Analysis of Variance (ANOVA) was conducted, using a model that included the experimental factors of ethanol, glycerol, and caffeic acid and all interactions. Neither individual panelists nor the replicates were included in this model, as panelists agreed with one another and were consistent across the three replicates. ANOVA was run on all attributes, using 30 s time bins, and results are shown in Table A1.

Generally, varying the compositional factors of ethanol, glycerol, and caffeic acid, led to both direct and indirect changes in sensory perception. Besides the expected direct effects on the taste and mouthfeel attributes of bitter, sweet, and warm/hot, we also observed indirect effects on all three flavor attributes of apple, pear, and citrus, due to changes in ethanol and glycerol levels. Changing levels of caffeic acid did not lead to any significant differences in temporal perception, most likely due to the comparatively small differences between the high and low level in caffeic acid.

Overall, varying ethanol levels led to most changes in all three modalities (flavor, taste, mouthfeel): for both apple and citrus flavor, the different ethanol levels significantly affected perception of these flavor attributes for the first 30 and 60 s, respectively. From Figure 1, we can deduce that increasing ethanol levels led to a decrease in apple and citrus flavors. Additionally, citrus flavor also showed a significant ethanol effect from 120 to 149 s. For sweet taste, similar to apple flavor, changing ethanol levels significantly affected the first 30 s of evaluation, indicating potential cross-modal correspondence of apple flavor and sweet taste perception. Samples lower in ethanol were perceived as having more sweetness and apple flavor (Figure 1).

In contrast, bitter taste, as expected, was affected by varying ethanol level in a more lasting manner, with a significant ethanol effect for nearly 2/3 of the evaluation period, from 0 to 119 s. Ethanol also affected, as expected, the perception of warm/hot mouthfeel, showing a significant contribution of ethanol to that perception for the complete evaluation period, except for a brief pause between 120 and 150 s. For both attributes, higher ethanol levels led to increased citation frequencies (Figure 1).

The second most common effect was found for the glycerol factor, significantly affecting pear flavor perception from 30 to 59 s, leading to increased pear flavor with increasing glycerol. This observation could be explained by another cross-modal correspondence of a retronasal aroma of pear and sweet taste elicited by glycerol. Glycerol significantly affected sweet taste perception for the first 120 s, while for bitter taste, different glycerol levels affected this taste from 0 to 89 s. Last, astringent mouthfeel showed a significant glycerol effect for the first 60 s. Generally, higher glycerol content led to increased sweet taste frequencies and lower frequencies of bitter taste and astringent mouthfeel.

Of particular interest for this study were interactions between the compositional factors—the perception of bitter taste was significantly affected by the interaction of ethanol with glycerol for the last 30 s of evaluation, from 120 to 180 s, where, for the 14% ethanol samples, greater differences were observed for the high vs. low glycerol samples, compared to the 10% ethanol samples, where both glycerol levels were perceived as very similar (Figure 1).

## 4. Discussion

This study demonstrated that major wine matrix components, namely, ethanol, glycerol, and caffeic acid, affect the dynamic perception of sensory attributes, both directly as well as interactively. Some factors and interactions even affect multiple sensory attributes, indicating intra-modal taste-taste and cross-modal flavor–taste interactions.

Overall, ethanol had the greatest effect of all compositional factors on the temporal perception of the model wines, both in number of affected attributes (apple and citrus flavor, sweet and bitter taste, and warm/hot mouthfeel) as well as how long that effect lasted.

Varying glycerol levels led to significant effects for pear flavor, sweet and bitter taste, and astringent mouthfeel; compared to ethanol, the effects of glycerol were lower, both in number of attributes affected as well as duration. Last, the effect of caffeic acid variation was not found to be significant for any attributes at any time during the evaluation.

Taking these results together, the observed effects could be explained by both inter- and intra-modal phenomena: for example, glycerol had a significant effect on both pear flavor and sweet taste during the 30–59 s time segment. Both pear flavor and sweet taste were cited more frequently when glycerol was higher. Prior studies indicate that glycerol, at levels typically found in wines (7–10 g/L), affects sweet taste [37,38].

While glycerol by itself has been described as sweet tasting [37], the changes in pear flavor perception can only be explained by cross-modal perceptual interactions, as none of the experimental factors in the study should have caused an increase in fruity, e.g., pear, flavors. Hort and Hollowood’s findings from 2004 suggest that the perception of fruity flavors is directly linked to the sweetness level [39], providing further evidence for cross-modal interaction between glycerol-induced sweet taste and increased apple flavor perception. The close positions of sweet taste to the fruit flavors of apple and pear would also suggest cross-modal interactions, where fruits and their flavors are learned to be associated with sweet taste [39].

Glycerol also significantly affected bitter taste: while sweet taste increased with increasing levels of glycerol, bitter taste decreased, suggesting taste-taste suppression. Jones et al. [3] similarly reported bitter taste suppression by glycerol, most likely due to the sweetness that it provides.

Taste-taste interaction could also explain the effects that ethanol has on both bitter taste and sour taste. For the high ethanol samples, higher frequencies of bitter taste and warm/hot mouthfeel were found; ethanol has been shown to elicit both bitter taste and a warming mouthfeel [40]. However, we also found that sour taste frequencies were low at high ethanol levels. We believe that the bitter taste elicited by the high ethanol levels is suppressing sour taste perception, similarly to prior literature for ethanol solutions [41] and white wines [42] where sour taste was suppressed by the bitterness of increased ethanol levels. Similar to bitter-sour interaction effects, sweet taste perception is known to be suppressed by bitter stimulants and vice versa [16]. For solutions of ethanol and sucrose, Martin and Pangborn [41] report increased sweetness ratings at 8% (*v/v*), but this effect flips at the higher tested concentrations of 12 and 24% (*v/v*). In line with the argument by Lanier et al. [43], lower levels of ethanol are perceived as sweet, therefore, the observed opposite positioning of sweet and sour taste to bitter taste and warm/hot mouthfeel could be explained by both direct (i.e., sweet taste receptor activation by low ethanol levels), as well as indirect effects (i.e., sweet-bitter mixture suppression).

Ethanol also seems to be responsible for cross-modal flavor–taste interactions at the beginning of the temporal evaluation, namely, a decrease in apple flavor citation frequency at increased ethanol levels, accompanied by increased bitter taste frequencies. A similar, cross-modal fruit flavor suppression by increased bitter taste perception was reported for red model wines [44].

Addressing one of our initial hypotheses, the use of a temporal evaluation method, namely, TCATA, in this study provides novel insight into wine matrix effects on sensory perception. As mentioned in the introduction, wine sensory attributes have traditionally been described using classical descriptive analysis. Using TCATA as an evaluation method does unveil similarities to DA but it also picks up on temporal changes that classical DA cannot.

One similarity that can be seen with both static DA and dynamic TCATA is in the astringent mouthfeel attribute: Our earlier study characterized the same model wine samples by DA. Comparing the DA results [29] to the findings of the TCATA study, both uncovered a significant effect of ethanol and glycerol on astringent mouthfeel, with counter-acting effects: Increasing levels of ethanol led to more astringency and increasing levels of glycerol led to less astringency. The TCATA results show that these significant effects were perceived immediately, within the first 5 s of evaluation. This is consistent with the DA results, most likely due to the fact that DA ratings were made immediately after tasting the samples.

However, for factors and interactions that had a significant effect later in evaluation, for example, past 60 s, the DA results do not match up well with the TCATA results. As an example, there are significant differences in warm/hot mouthfeel and bitter taste well past 60 s. This is not surprising as these attributes can linger for quite a while. These temporal differences in mouthfeel and taste attributes were generally caused by ethanol, glycerol, and the interaction between the two factors. These significant effects are not uncovered using DA, as the significant differences may not appear until at least a minute after the DA ratings are already made. As we know that wine is a complex sample with sensory qualities that change over time, a temporal method of evaluation must be used to capture these changes and completely describe the sample.

While we do see similarities between DA and TCATA results, there are also instances where they do not agree. These discrepancies mainly occurred for the taste attributes, but not the flavor or mouthfeel attributes. For example, the DA revealed significant differences in citrus flavor due to the interaction between ethanol and glycerol [29]. From the TCATA results, however, significant differences in citrus flavor perception were caused by ethanol, and in addition, these differences were seen for the first 60 s and then again towards the end of the evaluation (120–149 s).

Besides the difference in duration of assessment (one minute maximum in DA vs. up to three minutes in TCATA), these discrepancies could also be method-inherent. For example, it could be that panelists could take a couple of seconds to select present attributes at the start of TCATA evaluation. This could mean that differences that are present from the start are not immediately picked up because the attributes are not immediately selected. However, as flavor differences are perceived for a longer period (over 30 s), this delay in attribute selection may not be a great contributing factor. Secondly, differences could also be explained by the “grainier” nature of data collection in TCATA, compared to DA. DA results are nominal data of intensity ratings of sensory attributes, while TCATA results are binary (present-not present), that get converted into frequencies across participants. While previous studies have shown that intensity data is generally highly correlated with frequency data [19], the two methods will not produce identical results. Comparing DA and TCATA, the latter is a less refined method as it allows for only two outcomes (present or absent), while a scale, as used in DA, allows for more nuanced evaluations, and thus, smaller changes might not be picked up by TCATA.

In summary, it was found that changing major wine matrix components, such as ethanol and glycerol, affect temporal sensory perception of flavor, taste, and mouthfeel attributes both in direct and indirect manner. Taste-taste as well as cross-modal flavor–taste interactions were most likely the cause for our findings, providing further evidence of the complex nature of wine perception. Future work will validate these results in a real white, hybrid wine in order to fully understand the complex interactions between wine matrix components and aroma compounds.

## Figures and Tables

**Table 1:** (**a**) Major wine components and levels used in the model wines, and (**b**) composition of the aroma fraction, prepared in 200 proof ethanol, which was added to all model wines (1 mL/500 mL).

(**a**)	**Component**		**Levels**			
	Ethanol (% *v/v*)		10	12		14
	Glycerol (g/L)		5		15	
	Phenolics (mg/L, caffeic acid)		20		60	
	pH (−)		3.5			
	Protein (g/L, bovine serum albumin)		0.5			
	Titratable acidity (g/L, potassium hydrogen tartrate)		7.5			
(**b**)	**Compound**	**Concentration (μg/L)**	**Compound**		**Concentration (μg/L)**	
	Ethyl Acetate	50,000	Ethyl Hexanoate		1250	
	Acetic Acid	40,000	Hexyl Acetate		790	
	Phenethyl Alcohol	14,000	Ethyl Butanoate		450	
	Octanoic Acid	9100	Phenethyl Acetate		450	
	Hexanoic Acid	8100	β-Damascenone		415	
	Isoamyl Acetate	3200	Linalool		165	
	Ethyl Octanoate	1700	Geraniol		15	
	Ethyl Decanoate	1600				

**Table 2:** References used for training of sensory attributes. All standards were prepared in 10% (*v/v*) ethanol (Decon Labs, King of Prussia, PA), unless otherwise noted.

Attribute	Reference Standard
Apple	1 green apple jolly rancher (Hershey’s, Hershey, PA);10.0 g fresh Granny Smith apple (Wegmans, State College, PA) in 30 mL
Pear	30.0 g fresh green pear (Wegmans) in 20 mL; 5.0 g fresh Granny Smith apple; 20.0 g of fresh green pear in 20 mL
Citrus	3 × 2 cm fresh orange peel (Wegmans) in 20 mL; 3 × 2 cm fresh orange peel; 3 × 2 cm fresh lemon peel (Wegmans); 2 × 1 cm fresh lime peel (Wegmans) in 20 mL
Sour	1.5 g/L tartaric acid (>99.7%, Sigma-Aldrich, St. Louis, MO, USA) in deionized water
Sweet	30 g/L sucrose (Domino Foods Inc., Yonkers, NY, USA) in deionized water
Bitter	0.8 g/L caffeine (Sigma-Aldrich) in deionized water
Astringent	1.5 g/L alum (McCormick, Hunt Valley, MD) in deionized water
Warm/Hot	6% (*v/v*) ethanol (Decon Labs) in deionized water

**Table 3:** Panelist repeatability for the 12 panelists and panel agreement for three sensory attributes at 10% and 14% (*v/v*) ethanol.

Panelist	Citrus Flavor		Warm/Hot Mouthfeel		Sweet Taste		Repeatability
	10%	14%	10%	14%	10%	14%	
1	0.48	0.46	0.46	0.60	0.16	0.16	0.78
2	0.73	0.78	0.54	0.62	0.79	0.80	0.85
3	0.65	0.75	0.68	0.73	0.75	0.78	0.82
4	0.75	0.76	0.65	0.75	0.79	0.82	0.88
5	0.76	0.69	0.61	0.74	0.72	0.82	0.77
6	0.74	0.79	0.64	0.58	0.79	0.82	0.87
7	0.74	0.74	0.67	0.75	0.79	0.78	0.75
8	0.71	0.77	0.65	0.72	0.77	0.80	0.86
9	0.53	0.78	0.55	0.67	0.76	0.75	0.81
10	0.73	0.79	0.61	0.75	0.8	0.76	0.86
11	0.71	0.76	0.64	0.75	0.67	0.82	0.81
12	0.76	0.75	0.65	0.75	0.67	0.57	0.79

## Appendix A

**Table A1:** Degrees of freedom (DF) and *F*-values for the compositional factors ethanol (E), caffeic acid (C), glycerol (G) and all interactions, for each sensory attribute and 30 s time bins (bold = *p* ≤ 0.05). Mean squares are listed for the residuals (Res.).

	**Apple Flavor**								**Citrus Flavor**							
**Factor**	**E**	**C**	**G**	**E:C**	**E:G**	**C:G**	**E:C:G**	**Res.**	**E**	**C**	**G**	**E:C**	**E:G**	**C:G**	**E:C:G**	**Res.**
*DF*	*2*	*1*	*1*	*2*	*2*	*1*	*2*	*420*	*2*	*1*	*1*	*2*	*2*	*1*	*2*	*420*
0–29 s	**3.56**	0.44	1.52	0.63	0.22	0.20	0.36	106.22	**6.38**	0.69	3.72	**3.80**	0.59	1.15	0.36	93.32
30–59 s	0.09	0.55	0.94	0.12	0.98	0.02	0.19	179.29	**3.16**	0.39	0.07	0.48	0.11	0.20	0.09	173.18
60–89 s	0.37	0.14	0.87	0.10	0.45	0.09	0.17	152.82	0.48	0.00	0.27	0.09	1.08	0.07	0.02	139.48
90–119 s	0.03	0.17	1.40	0.31	0.13	0.28	0.25	105.53	1.59	0.03	0.69	0.07	0.21	0.01	0.19	79.92
120–149 s	0.03	0.08	1.23	0.23	0.04	0.15	0.91	80.89	**4.24**	0.13	1.28	0.12	0.02	0.36	0.16	48.23
150–180 s	0.06	0.01	0.81	0.21	0.26	0.03	0.80	76.69	2.51	0.29	1.79	0.42	0.39	0.00	1.88	23.27
	**Pear Flavor**								**Sweet Taste**							
**Factor**	**E**	**C**	**G**	**E:C**	**E:G**	**C:G**	**E:C:G**	**Res.**	**E**	**C**	**G**	**E:C**	**E:G**	**C:G**	**E:C:G**	**Res.**
*DF*	*2*	*1*	*1*	*2*	*2*	*1*	*2*	*420*	*2*	*1*	*1*	*2*	*2*	*1*	*2*	*420*
0–29 s	0.93	0.93	10.98	1.47	0.52	0.19	1.32	107.51	**6.75**	0.43	**25.64**	0.02	0.26	0.38	0.21	97.64
30–59 s	0.89	0.37	**4.34**	1.22	0.77	0.37	1.13	178.36	1.38	0.00	**11.73**	0.44	0.64	1.04	0.90	173.24
60–89 s	1.02	0.21	2.22	0.05	0.95	0.63	0.53	190.24	0.68	0.00	**8.51**	0.34	0.24	0.87	0.71	139.77
90–119 s	1.54	0.49	2.00	0.17	0.28	0.62	0.11	149.76	0.316	0.03	**5.033**	0.11	1.02	0.15	0.176	103.34
120–149 s	1.21	1.73	1.14	0.13	0.56	0.12	0.15	119.15	0.23	0.17	0.954	0.17	0.25	0.07	0.038	78.63
150–180 s	0.20	0.81	0.29	0.01	0.42	0.35	0.06	100.94	0.00	0.00	0.03	0.01	0.00	0.00	0.01	75.90
	**Sour Taste**								**Bitter Taste**							
**Factor**	**E**	**C**	**G**	**E:C**	**E:G**	**C:G**	**E:C:G**	**Res.**	**E**	**C**	**G**	**E:C**	**E:G**	**C:G**	**E:C:G**	**Res.**
*DF*	*2*	*1*	*1*	*2*	*2*	*1*	*2*	*420*	*2*	*1*	*1*	*2*	*2*	*1*	*2*	*420*
0–29 s	2.16	2.44	0.546	0.11	0.08	0.83	0.117	80.78	**11.96**	0.03	**12.79**	0.39	0.52	0.06	0.83	88.17
30–59 s	0.26	0.62	0.035	1.05	0.5	0	0.432	165.24	**11.99**	0.24	**5.01**	1.18	1.33	1.77	0.94	154.39
60–89 s	0.08	0.02	0.096	1.03	0.08	0.05	0.257	146.44	**8.38**	1.41	**5.18**	0.65	0.51	0.17	0.91	170.78
90–119 s	1.72	0.12	0.146	0.21	0.27	0.1	0.062	81.98	**3.10**	1.17	2.73	0.34	1.64	0.37	0.51	102.08
120–149 s	0.66	0.02	0.036	1.27	0.37	0.5	0.367	50.27	2.03	1.25	0.72	0.12	**5.12**	0.02	0.05	25.40
150–180 s	0.03	0.64	0.749	1.16	0.12	0.12	0.877	31.51	1.06	0.22	1.05	1.57	**3.50**	2.92	0.22	5.07
	**Astringent Mouthfeel**								**Warm/Hot Mouthfeel**							
**Factor**	**E**	**C**	**G**	**E:C**	**E:G**	**C:G**	**E:C:G**	**Res.**	**E**	**C**	**G**	**E:C**	**E:G**	**C:G**	**E:C:G**	**Res.**
*DF*	*2*	*1*	*1*	*2*	*2*	*1*	*2*	*420*	*2*	*1*	*1*	*2*	*2*	*1*	*2*	*420*
0–29 s	1.87	0.08	**4.71**	0.48	0.22	0.07	0.12	93.36	**19.16**	0.22	0.03	0.55	0.73	0.84	0.45	71.65
30–59 s	1.42	0.02	**4.24**	0.08	0.10	0.04	1.22	181.02	**24.99**	0.62	1.578	0.05	0.86	0.47	0.44	78.97
60–89 s	0.38	0.04	0.10	0.02	0.09	0.02	0.05	202.58	**20.59**	0.37	3.08	0.18	0.17	0.10	0.55	142.09
90–119 s	0.21	0.09	0.42	0.32	0.19	0.14	0.34	182.60	**8.52**	0.10	0.00	0.30	0.11	0.36	0.42	185.57
120–149 s	0.22	0.74	0.15	0.26	1.06	0.32	0.93	155.33	2.43	0.08	0.43	0.08	0.08	0.37	1.02	154.26
150–180 s	0.14	0.64	0.00	0.02	0.41	0.19	0.88	109.71	**4.67**	0.22	0.05	0.12	0.25	1.00	0.41	93.19
